# Auditory and visual cueing modulate cycling speed of older adults and persons with Parkinson’s disease in a Virtual Cycling (V-Cycle) system

**DOI:** 10.1186/s12984-016-0184-z

**Published:** 2016-08-19

**Authors:** Rosemary Gallagher, Harish Damodaran, William G. Werner, Wendy Powell, Judith E. Deutsch

**Affiliations:** 1 Department of Physical Therapy, School of Health Professions, New York Institute of Technology, Old Westbury, NY USA; 2 Rivers Lab, Department of Rehabilitation and Movement Sciences, School of Health Professions, Rutgers University Newark, Newark, NJ USA; 3 School of Creative Technologies, University of Portsmouth, Portsmouth, UK

**Keywords:** Virtual environments, Virtual reality, Motor learning, Cueing, Bicycling, Exercise intensity, Parkinson Disease, Older adults

## Abstract

**Background:**

Evidence based virtual environments (VEs) that incorporate compensatory strategies such as cueing may change motor behavior and increase exercise intensity while also being engaging and motivating. The purpose of this study was to determine if persons with Parkinson’s disease and aged matched healthy adults responded to auditory and visual cueing embedded in a bicycling VE as a method to increase exercise intensity.

**Methods:**

We tested two groups of participants, persons with Parkinson’s disease (PD) (*n* = 15) and age-matched healthy adults (*n* = 13) as they cycled on a stationary bicycle while interacting with a VE. Participants cycled under two conditions: auditory cueing (provided by a metronome) and visual cueing (represented as central road markers in the VE). The auditory condition had four trials in which auditory cues or the VE were presented alone or in combination. The visual condition had five trials in which the VE and visual cue rate presentation was manipulated. Data were analyzed by condition using factorial RMANOVAs with planned t-tests corrected for multiple comparisons.

**Results:**

There were no differences in pedaling rates between groups for both the auditory and visual cueing conditions. Persons with PD increased their pedaling rate in the auditory (F 4.78, *p* = 0.029) and visual cueing (*F* 26.48, *p* < 0.000) conditions. Age-matched healthy adults also increased their pedaling rate in the auditory (*F* = 24.72, *p* < 0.000) and visual cueing (*F* = 40.69, *p* < 0.000) conditions. Trial-to-trial comparisons in the visual condition in age-matched healthy adults showed a step-wise increase in pedaling rate (*p* = 0.003 to *p* < 0.000). In contrast, persons with PD increased their pedaling rate only when explicitly instructed to attend to the visual cues (*p* < 0.000).

**Conclusions:**

An evidenced based cycling VE can modify pedaling rate in persons with PD and age-matched healthy adults. Persons with PD required attention directed to the visual cues in order to obtain an increase in cycling intensity. The combination of the VE and auditory cues was neither additive nor interfering. These data serve as preliminary evidence that embedding auditory and visual cues to alter cycling speed in a VE as method to increase exercise intensity that may promote fitness.

## Background

Exercise is essential for persons with Parkinson’s disease (PD) and older adults to maintain optimal health [1]. However, barriers to exercise such as poor health and unsafe exercise environments [2, 3] can affect motivation and result in an overall decrease in physical activity [4]. Therefore there is a need to find safe, available, and engaging exercise programs for these populations.

The American College of Sports Medicine recommends that adults of all ages, including those with chronic disease or disabilities, engage in continuous moderate or vigorous exercise on a regular basis to ensure optimal health [1]. Regular physical activity is associated with numerous health benefits in all adults including improvements in cardiovascular, motor, and cognitive function [5–10]. In persons with PD, exercise may also be neuroprotective, and help decelerate the disease process [5, 11, 12].

Many factors, such as exercise timing, type, and intensity, determine the extent of benefit of exercise [1, 5]. High intensity exercise when compared to low intensity exercise has been shown to promote greater cardiovascular, metabolic and musculoskeletal health for older adults and improved motor function for persons with PD [13]. Specifically for persons with PD, high intensity treadmill training studies have demonstrated improvements in muscle activation, motor function, mobility, gait, and quality of life [14–16], as well as evidence of neuroplastic changes when cognitive challenges were introduced [17]. Importantly, these studies also show that persons with PD can tolerate exercise at high intensities [14, 17].

Stationary cycling is a viable form of aerobic exercise that is safe and commonly used in healthy and patient populations, including persons with PD, to improve cardiovascular fitness while minimizing joint stress [9, 18]. In fact, people with PD can often ride a bike even after their ability to walk is compromised [19].

High intensity cycling studies in persons with PD are based on studies in animal models that show high intensity exercise improves motor function, and is also neuroprotective [20, 21]. Early studies by Ridgel and colleagues investigated ‘forced-use’, or high intensity cycling that employed a tandem bicycle to force a pedaling rate an average 30 % faster than the voluntary pedaling rate of participants with PD. Mitigation of symptoms such as tremor, rigidity, and bradykinesia were found [20]. More recent studies found that a single session of high intensity active assisted cycling reduced tremors and improved bradykinesia in persons off medication [21]. In a 2015 study, three sessions of high intensity cycling improved motor symptoms in not only the lower, but the upper extremities as well. In addition, a decrease in Timed Up and Go scores brought participants from a high fall risk to a no fall risk range [22]. These results suggest that pedaling at a high rate may improve symptoms of PD and supports the use of high intensity exercise as an alternative to medication to manage symptoms.

Virtual environments (VE) are simulations of real world environments that provide complex multisensory information to the user [23, 24] in a safe, engaging, and motivating context [25]. Virtual environments and serious games (using game theory and game mechanics to address a serious purpose such as education or rehabilitation, in contrast to recreation) have been successful in improving mobility and physical activity in healthy people and persons with PD [26–29]. Persons with PD have difficulty generating appropriate effort when moving and show reduced amplitude of movement compared to their healthy counterparts [30]. External cues may compensate for defective internal mechanisms that cause these deficiencies and result in more normal execution of movement [12]. Virtual environments can be tailored to incorporate compensatory techniques such as cueing, and motor learning principles such as the provision of feedback, repetition, and high intensity training. For example, an 8-week training program using a cycling VE developed by Deutsch et al, successfully improved fitness levels in people post-stroke [31].

External cueing, both auditory and visual, have been found to positively affect motor behavior in healthy people and in persons post-stroke and with PD not only in real-world settings [32–34] but also in VEs [27, 35]. An important consideration when studying the influence of a VE on motor behavior is the role of optic flow, the visual perception of movement produced by a person’s own actions [36]. Optic flow provides powerful information that influences the speed and direction of movement during walking in older adults [37–40], in persons post-stroke [41], and persons with PD [38, 42], and also in cycling in older adults [43, 44] and persons post-stroke [45].

Visual cueing in a VE has been shown to modulate and be independent of optic flow [29]. Van Wegen et al. investigated the influence of visual cues on stride frequency and walking velocity in healthy older adults and people with PD on a treadmill [29]. Due to an increased reliance on vision in persons with PD [46, 47], the possibility of a suppressive effect when the VE was presented with the visual cue (a rhythmic flashing light) existed. However, participants were able modulate their stride frequency when the visual cues were presented with the VE, indicating that the presence of the VE did not interfere with the ability to respond to the external cues [23].

Coupling auditory cues and optic flow in a VE has been studied in walking [48]. Powell et al. sought to determine if auditory cueing presented in a VE would influence gait speed in healthy adults while walking on a treadmill [48]. The VE and auditory cues were presented alone and in combination; three audio cue rates were used: 75, 100 and 125 % of baseline speed. The addition of optic flow to the fast and slow audio cue conditions resulted in a significant decrease in walking speed compared to the audio cue only condition, suggesting an increased demand on cognitive resources for motor execution in the presence of a VE. The influence of auditory or visual cueing embedded in a cycling VE has not been investigated. Therefore, it is unknown if there will be a suppressive or additive effect. Investigating these potential interactions is one of the purposes of this study.

In summary, VEs provide clinicians with a tool to train and rehabilitate persons with PD and healthy older adults, and may serve to optimize motor learning and fitness in a rehabilitation setting. However, despite the evidence to support the use of VEs to improve gait and for exercise promotion, there is no direct evidence to support the efficacy of external cueing embedded in a virtual cycling environment for fitness and activity promotion. Therefore, an evidence-based virtual cycling environment embedded with auditory and visual cues was developed to determine if pedaling rate would increase in persons with PD and age-matched healthy older adults. While between-group comparisons were measured, our primary interest was comparisons within-groups. We also sought to determine if there would be interference or an additive effect between auditory cues and the VE, and if persons with PD would show a stronger response than the age-matched healthy adults to the visual cues. Secondarily we confirmed the validity of the VE by measuring if the percent increase in cycling was proportional to the augmented cues.

Based on evidence from the literature, we hypothesized that both groups would respond to the auditory and visual cueing by increasing pedaling rate, and that age-matched healthy adults would pedal at a faster rate under all conditions compared to persons with PD. We also hypothesized that persons with PD would respond more strongly to visual cues than age-matched healthy older adults. When auditory and visual cueing were combined, we proposed a non-directional hypothesis due to the possibility of either an interference or additive effect. We also expected that the increase in pedaling rate for both groups would be proportionate to cue rate.

## Methods

### Study design

This study used a cross sectional design. Eligible participants consisted of persons with PD and age-matched healthy adults. The Institutional Review Board at the New York Institute of Technology and Rutgers University School of Health Professions approved this work. All participants provided written informed consent prior to participation.

### V-CYCLE system

The virtual reality cycling system V-CYCLE, consists of an evidenced-based custom designed VE, computer, projector display of the VE on a screen, desktop speakers, upright stationary bicycle, revolutions per minute (RPM) sensor, and heart rate monitor.

### Unity game design

The VE was built specifically for this study using the free version of Unity 4.3™. Factors embedded in a VE can facilitate or hinder motor behavior [49]. Therefore elements in the V-CYCLE environment were chosen after careful review of the literature and based on their ability to influence self-perception of motion.

- **Field of view**: a wide field of view incorporates visual cues in the periphery, thereby improving perception of self-motion and immersion. The ideal field of view is between 80 and 200° [49]. The field of view in the V-CYCLE environment was 80°.
- **Spatial frequency between objects**: Manipulating the spatial frequency between objects in the environment gives the user a sense of moving faster or slower through the environment [24, 50]. We decreased the spatial frequency between the central road markers (our visual cue) from a real-world distance apart to a 20 % faster presentation rate.
- **Color contrast and texture:** A high color contrast and the inclusion of texture in the environment improve the user’s self-perception of motion [51]. We ensured a high color contrast between the road, sky and grass, and movement of the foliage supplied texture.
- **Scale of objects:** Objects scaled to real-world proportions influence self-perception of motion [34, 49]. The objects in our environment were scaled to real-world proportions. For example, 6' in the real world = 3' in our VE.

**Fig. 1:**
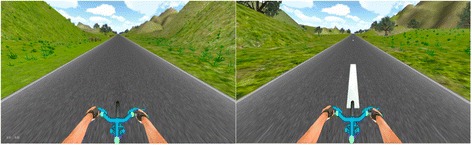
The VE without (L) and with (R) road markers, which are the visual cues (VE + VC). Road markers were presented at the baseline cycling rate of the participant then increased by 20 %

The scenery, consisting of a road, mountains, trees and sky, was designed using the default terrain editor of Unity 4.3 with a first person perspective view (Fig. 1). The goal of the design process was to create an open straight road surrounded by mountains with an adequate field of view and variability in the scenery.

The models and avatars used during the design were purchased or downloaded from the Unity asset store. Rendering was done using the built in renderer for terrain, and Skybox for the clouds and sky. The input manager was used to accept keyboard controls for pausing, quitting, and manual override functions for control of the avatar. Scripts within Unity were written in C++ to customize and have control over the VE during the trial. The RPM (Wahoo RPM sensor) and heart rate (Polar HR7) data were collected and recorded independent of Unity using a Wahoo SDK and saved as a.CSV file. This file was used to read the pedal RPM data from the Wahoo sensor to control the speed of the rider. The linear distance covered by the bike/minute in the VE was calculated as (2π * radius of wheel) * RPM. The status of data collection and timer was controlled using a C++ script. The virtual environment utilizes the RPM data from the. CSV output file to control the speed of the avatar in the VE in which 6′ of pedaling corresponds to 3′ of distance in the VE.

### Auditory and visual cueing

Auditory cueing was provided by a metronome set at a rate 20 % higher than the cycling speed of the subject. The 20 % rate was based on the walking literature [52, 53] as well as preliminary trials performed by the investigators on three healthy and three persons with PD to determine a physiological upper limit of pedaling rate. Visual cueing was in the form of central road markers in the VE, scaled to represent a real road.

### V-Cycle set up

**Fig. 2:**
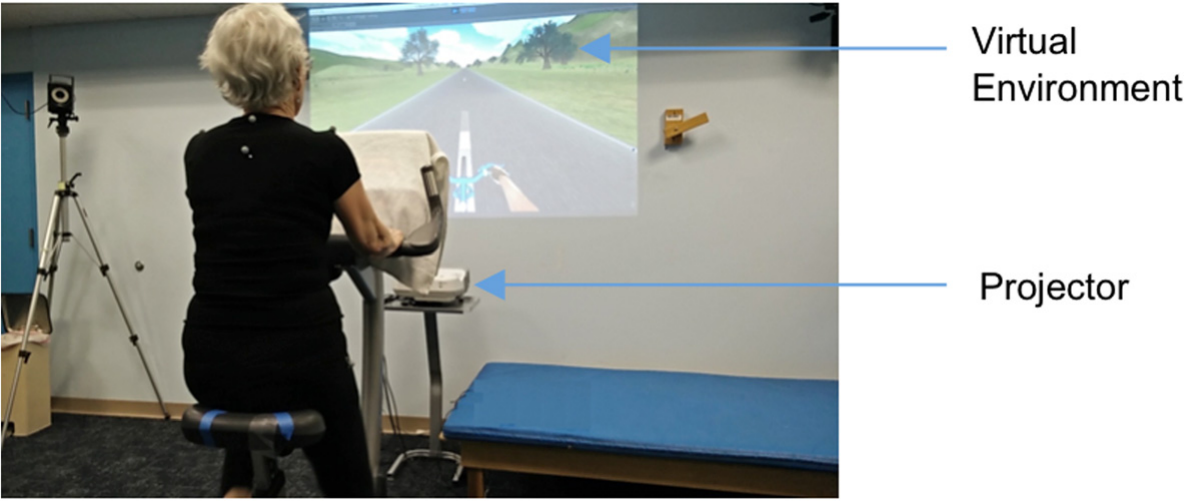
V-CYCLE System set up. The virtual environment displayed via a short throw projector, was projected onto a flat wall approximately 5′ in front of the participant

An upright stationary bicycle (Cybex model #750C) was used in this study. A Wahoo cadence sensor attached to the crank of the bike pedal measured the pedal RPM and transferred the data via Bluetooth™. An Epson (Model 485Wi) short throw projector was used to project the environment onto a flat wall, approximately 5 ft in front of the bicycle, resulting in an equivalent screen size of 94-in. (43 X 83 in.) with a horizontal field of view of 80° (Fig. 2). A pair of Logitech desktop speakers connected to an IPhone metronome application was used for trials with audio cueing.

### Participants

Twenty-eight participants, 15 people with PD (66.3 +/− 9.6 years; Hoehn &Yahr (H&Y) stages II and III) [54] and 13 age-matched healthy adults (66.7 +/− 9.1, years), voluntarily participated in the study. Participants were recruited through flyers, referral, and exercise groups. Age-matched healthy adults were spouses or friends of participants with PD. Telephone or in-person interviews were used to screen for eligibility. Participants were included if they were 50 to 85 years inclusive, able to ride a stationary upright bicycle and had a Montreal Cognitive Assessment (MoCA) [55] score >/= 24. Participants with PD were included if they were diagnosed by a neurologist as having PD and were in stage 2–3 H&Y [54]. Participants were excluded if they had: 1. severe hearing or visual deficit including color blindness; 2. history of stroke, traumatic brain injury or neurological disorder other than PD; 3. unstable medical condition including musculoskeletal disorders such as severe arthritis, knee surgery, hip surgery; or any other condition that the investigators determine would impair the ability to ride a stationary bicycle; 4. medical or musculoskeletal contraindications to exercise. Participants with PD were excluded if they had incapacitating tremors or dyskinesias that would limit ability to ride a stationary bicycle.

### Procedure

Participants attended two testing sessions lasting approximately 1 h each. The first session characterized the participants by measuring: age, gender, mental status, and lower extremity range of motion. Participants with PD were clinically rated by a trained examiner on the H&Y scale [54] and the Motor subsection (part III), of the Unified Parkinson’s Disease Rating Scale (UPDRS) [56].

The second session consisted of the bicycling protocol. Participants were seated on the bicycle with the seat height adjusted between 100 % and 110 % of the length from the greater trochanter to the floor (measured without shoes) [57]. After a 5-min warm-up, participants performed 9 trials (1 min each) of cycling divided into two blocks, Auditory (4 trials) and Visual (5 trials) (See Tables 1 and 2 for the description of trials). Each block included a baseline condition (cycling without a VE or cues) to ensure that pedaling rate changes were assessed relative to each block. Block order was counterbalanced between participants. To ensure the same frame of reference from one trial to the next, the order of trials was maintained within each block. This method of trial presentation has been used in similar studies [29].

**Table 1:** Auditory cueing: description of trials

Trial	Instructions to participant
Baseline	*Look ahead of you. Start pedaling until you reach a comfortable speed.*
AC	*Look ahead of you. Match your cycling speed to the metronome.*
VE	*Look ahead of you at the road.*
AC + VE	*Look ahead of you at the road. Match your cycling speed to the metronome*.

Baseline = no VE, no cueing, *VE* virtual environment without auditory cues, *AC* auditory cues without a VE

**Table 2:** Visual cueing: description of trials

Description	Instructions to participant
Baseline	*Look ahead of you.*
	*Ride at a comfortable pace.*
VE	*Look ahead of you at the road.*
VC	*Look ahead of you at the road.*
VC 20 % faster	*Look ahead of you at the road.*
VC 20 % faster with instruction	*Look ahead of you at the road. Try to decrease the gray space between the markers.*

Baseline = no VE, no cueing, *VE* virtual environment, *VC* visual cues, 20 % faster (spacing between markers decreased by 20 % compared to previous trial)

The 1-min trial length was chosen to capture short-term changes in cycling behavior while minimizing the effects of fatigue on cycling rate. The Borg scale [58] was used as a rate of perceived exertion and was shown to participants immediately after completing a trial. Heart rate was monitored throughout. Readiness to continue to the next trial was determined when heart rate returned to no more than ten beats above the warm up rate. Rest between trials ranged from 1 to 3 min.

### Outcome measures

The primary outcome measure was pedaling rate measured as RPMs. Pedaling rate was continuously recorded via a Bluetooth cadence sensor attached to the crankshaft of the pedal. Average cadence over the 1-min trial was calculated and used for data analysis. The first 5 s of each trial were not included in the analysis to allow participants to stabilize their cycling rate.

### E. data analysis

Descriptive analyses were performed on patient characteristics: age, gender, cognitive status, disease stage, and motor assessment. Differences between groups for baseline characteristics were tested with independent t-tests. Means and standard deviations were calculated for RPM with an alpha level of 0.05 and corrected for multiple planned comparisons using a Bonferroni correction.

#### Auditory condition

A 2 × 5 (group x condition) repeated measures factorial ANOVA was conducted to determine between and within group differences for the auditory condition. The alpha level was corrected based on the following five planned comparisons: baseline to auditory cues, baseline to VE, baseline to auditory cues + VE, auditory cues to auditory cues + VE, VE to auditory cues + VE. To determine if the change in pedaling rate was proportional to the auditory cue rate (a 20 % increase) the percent change from baseline to each condition was calculated.

#### Visual condition

A 2 × 4 repeated measures factorial ANOVA was conducted to determine between and within group differences for the visual condition. The corrected alpha level in the visual condition was based on the following four planned comparisons: baseline to VE, VE to VE with visual cues, VE to VE with visual cues to 20 % faster visual cues, 20 % faster visual cues to VE with instruction. To determine if the change in pedaling rate was proportional to the visual cue rate (a 20 % increase) the percent change from baseline to each condition was calculated. IBM SPSS (Version 22) was used for all analyses.

## Results

### Participants

Fifteen persons with PD and 13 age-matched healthy adults participated in the study. There were no significant differences in age or cognitive status between the two groups (Table 3). Participants with PD were in stage 2 or 3 on the H&Y scale [54].

**Table 3:** Participant Characteristics (*N* = 28)

	Parkinson’s disease *n* = 15		Age-matched healthy *n* = 13	
Characteristic	Mean (SD)	Range	Mean (SD)	Range
Age (y) mean (SD)	66.3 (9.6)	50–80	66.7 (9.1)	50–81
Gender (M/F)	13/2	------	7/6	------
MoCA	26.3 (1.9)	24–29	27.1 (2.3)	24–30
H&Y	2.3 (0.5)	------	------	------
UPDRS-Motor	35.5 (14.2)	------	------	------

*MoCA* Montreal Cognitive Assessment, *H&Y* Hoehn and Yahr Scale

UPDRS III Unified Parkinson’s disease Rating Scale Part III Motor subsection

### Auditory condition

**Fig. 3:**
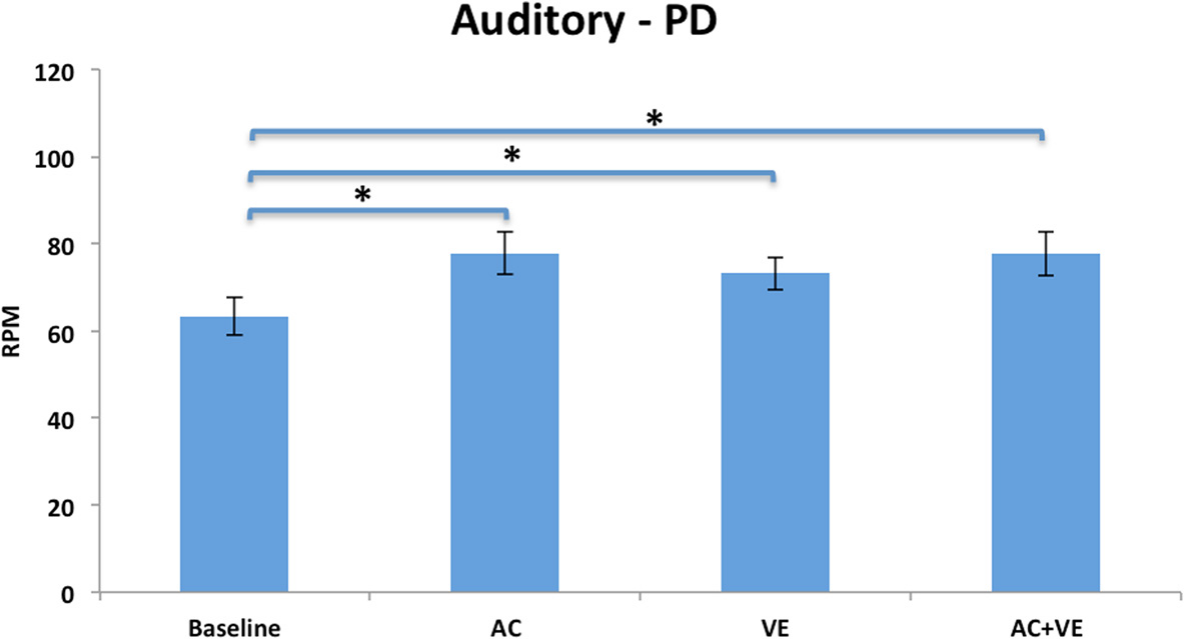
Auditory condition, PD: Mean (SE) RPMs. There was a significant increase in pedaling rate from baseline to all conditions. *Corrected alpha *p*=/<0.01

**Fig. 4:**
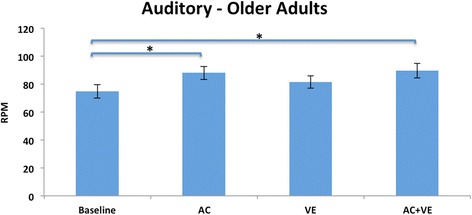
Auditory condition, Older Adults: Mean (SE) RPMs. There was a significant increase in pedaling rate from baseline with ACs and ACs combined with the VE. *Corrected alpha *p*=/<0.01

There was a significant main effect for cue, with no group or interaction effects. Age-matched healthy adults pedaled at a faster, albeit non-significant, rate than persons with PD in all conditions. Within group comparisons showed that both groups significantly increased their pedaling rate in the Auditory Condition (*F* = 24.72, df 1.7 *p* < 0.000). Compared to baseline, both groups increased their pedaling rate with the presentation of auditory cues; persons with PD, *p* < 0.000; age matched healthy adults, *p* < 0.000, and when auditory cues were presented with the VE; persons with PD: *p* < 0.000; age matched healthy adults *p* < 0.002. Persons with PD responded with an increase in pedaling rate to the presentation of the VE compared to baseline (*p* < 0.000) whereas the age-matched healthy adults did not (*p* = 0.017) (Figs. 3 and 4). Expected and observed changes in cycling speed are presented in Table 4.

**Table 4:** Auditory condition: expected and observed rpm changes

Condition	Parkinson’s disease		Age matched healthy adults	
	Expected (%)	Observed (%)	Expected (%)	Observed (%)
Baseline to AC	20	19	20	15
Baseline to VE	baseline	14	baseline	7
Baseline to AC + VE	unknown	18	baseline	17
AC to AC + VE	unknown	−1	unknown	2

baseline: increase from baseline rate but of unknown magnitude

unknown: additive effect (positive) or interference effect (negative)

### Visual condition

**Fig. 5:**
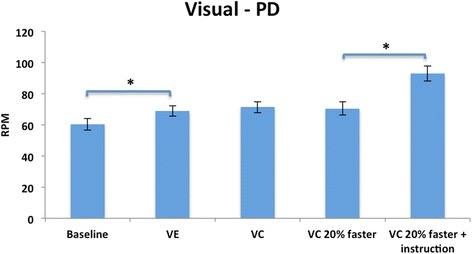
Visual condition, PD: Mean (SE) RPMs. There was a significant increase in pedaling rate between trials when the VE was added and when instructed to attend to the VC. *Corrected alpha, *p*=/<0.01

**Fig. 6:**
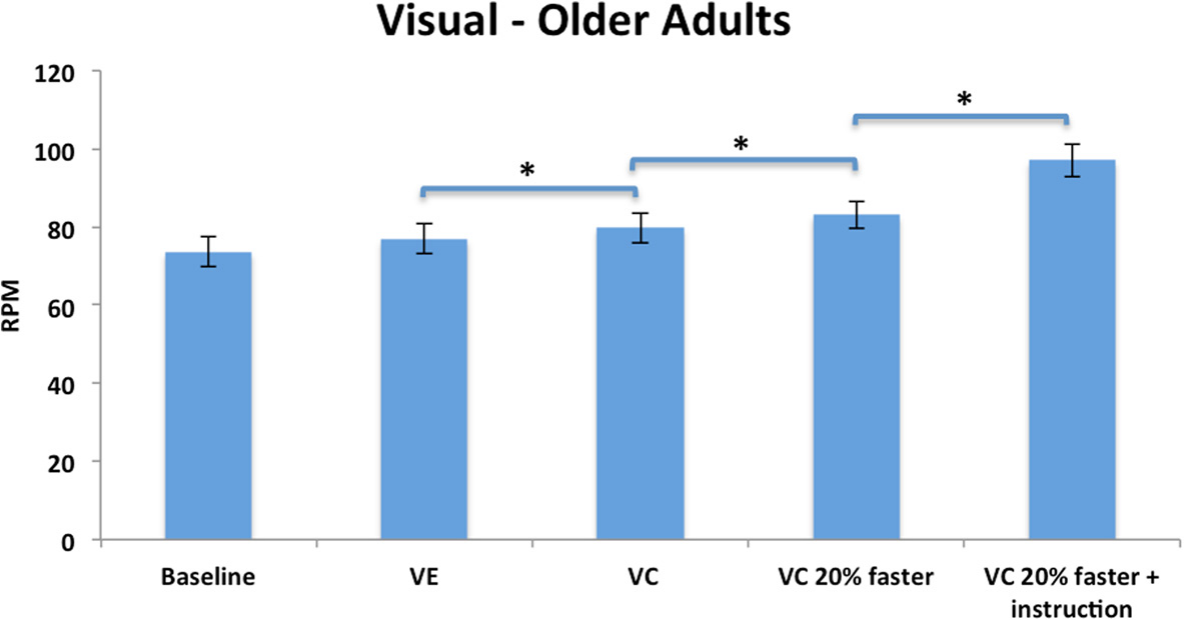
Visual condition, Older Adults: Mean (SE) RPMs. There was a significant increase in pedaling rate between trials when VC were added to the VE, when the VC were presented at a faster rate, and when instructed to attend to the VC. *Corrected alpha, *p*=/<0.01

There was a significant main effect for cue, with no group or interaction effects. Age-matched healthy adults pedaled at a faster rate than persons with PD in all conditions showing a trend toward significance (*F* = 4.00, df 1, *p* = .056). Within group comparisons showed that both groups significantly increased their pedaling rate (*F* = 40.69, df 4, *p* < 0.000). Comparisons within trials exclusive of baseline revealed that age-matched healthy adults increased their pedaling rate with each successive trial, but persons with PD increased their pedaling rate only when explicitly instructed to attend to the cues (*p* = 0.000) (Figs. 5 and 6).

The expected and observed changes in pedaling rate are presented in Table 5. The largest increase in pedaling rate for both groups (PD, 35 % and age-matched healthy adults, 25 %) was in the VE + VC 20 % with instruction condition.

**Table 5:** Visual Condition: Expected and Observed rpm changes

Condition	Parkinson’s disease		Age matched healthy adults	
	Expected (%)	Observed (%)	Expected (%)	Observed (%)
Baseline to VE	baseline	13	baseline	7
VE to VE + VCs	baseline	9	baseline	16
VE to VE + 20 % faster VCs	20	15	20	12
VE to 20 % faster VCs + instruction	20	35	20	25

baseline: increase from baseline rate but of unknown magnitude

## Discussion

The primary aims of this study were to develop and validate an evidenced based cycling VE (V-CYCLE) embedded with auditory and visual cues, and to determine if these cues influenced pedaling rate in persons with PD and age-matched healthy adults. Validity of the V-CYCLE was demonstrated as persons with PD and age-matched healthy adults modified their cycling behavior in response to the manipulations in the VE. While the groups did not differ, both groups increased their pedaling rate when compared to baseline.

### Auditory condition

The main findings in the auditory condition are that persons with PD and age-matched healthy adults increased their pedaling rate compared to baseline, and there was no interference effect when the auditory cues were presented with the VE. The increase in pedaling rate in both groups agrees with our hypothesis and aligns with the literature that healthy people can match their walking speed to an auditory cue [53, 59–61]. However, in contrast to the walking literature, there was no interference for either group when the VE and auditory cues where presented simultaneously [48].

The lack of interference found in this study may be attributed to a variety of reasons. First, elements in the periphery of a VE provide important peripheral cues that help increase immersion of the user in the environment. These cues also are also known to increase self-perception of motion [49]. The stimulus in this environment may have been weak due to a lack of peripheral cues and thus no interference effect was found. Alternatively, this finding may be explained by general differences between walking and cycling. In walking, one receives proprioceptive information regarding position while translating through space. This information contributes to muscle coordination and plays a role in the automaticity of walking [62]. During stationary cycling, there is no translation, and therefore proprioceptive inputs and response to these inputs may differ. A second explanation is that in cycling, angular momentum of the pedaling apparatus may keep the legs moving along [18] thereby off-setting any slowing down in pedaling rate from the VE. Lastly, there may have been an order effect due to the non-randomization of trials within each block. Participants heard the auditory cue in the first trial and may have continued to attend to it when the VE was presented.

### Visual condition

Both persons with PD and age-matched healthy adults increased their pedaling rate in most trials compared to baseline. Their patterns however, differed. Persons with PD significantly increased their pedaling rate with just the viewing of the VE but age-matched healthy adults did not. This is in agreement with our hypothesis and the literature that states persons with PD are more reliant on visual stimuli [46]. The stimulus of the optic flow with the VE alone stimulated a higher cycling rate for persons with PD and not age-matched healthy adults.

Persons with PD responded to the visual cues only when explicitly instructed to attend to the cues and not in the implicit cue conditions. The use of explicit instructions to augment motor performance is well demonstrated in the PD literature [5, 63]. Morris et al, in 1996 investigated the effects of visual cue training on the ability to walk to normal gait parameters [63]. Normalization of gait was found when subjects were explicitly instructed to attend to the markers, “step over the markers and walk to the end of the walkway”. Similarly, van Wegen et al., found that explicit instruction to attend to visual cues modulated stride frequency while maintaining walking velocity in persons with PD [29]. Our findings, and the evidence in the literature, have implications for adding explicit messages into a VE to increase the likelihood of achieving the target motor behavior.

As expected, age-matched healthy adults responded to progressively faster visual cues, while persons with PD did not. This may be because the increase in optic flow speed preferentially influenced pedaling rate in age-matched healthy adults. This finding is in agreement with the literature that states that decreasing the spatial frequency between objects in a VE gives the impression of moving faster through the environment [24, 50]. This finding also suggests that stimuli in the VE alone may not have been salient enough to produce a response in persons with PD. Alternatively, unless explicitly instructed to attend to a cue, persons with PD were not able to process the stimuli fast enough.

Contrary to our hypothesis, age-matched healthy adults did not pedal significantly faster than persons with PD in either the auditory or the visual condition. This may be explained in part by the high functioning persons with PD that were studied. The difference in the performance under the visual condition approached significance, with age-matched healthy adults pedaling faster than persons with PD. However, the percent change from baseline was greater for persons with PD.

#### Limitations

When designing a VE, embedded elements may facilitate or hinder motor behavior [49]. The following factors may have affected the degree of immersion that participants experienced and explain the lack of interference that is found in walking studies [48]. For example, the size of the field of view influences a participants’ degree of immersion and perception of self-motion [44, 49], which can limit the ability to appropriately respond to elements in the environment. The field of view in the V-CYCLE was 80°, which is at the lower limit of ideal size (80 to 120°) [49]. However, our VE was designed for use in a clinical setting where space may be limited.

Using a monoscopic rather than a stereoscopic projection may have influenced behavior of our participants. A stereoscopic projection provides separate images to each eye thereby increasing depth perception. This in turn increases self-motion perception and sense of immersion in the environment [49]. A monoscopic projection was chosen for this study because of its ease of use and lower cost, and therefore more amenable to the clinical setting.

The use of horizontal rather than vertical lines as a visual cue may have also influenced cycling behavior. Our simulation was adapted from the walking literature, which typically use lines oriented perpendicular to the walking progression [29, 63–65]. The visual cues in the V-CYCLE were oriented vertical to the scene to make the environment ecologically valid. Although the vertical orientation of the cues did not appear to limit performance, future designs may specifically test if visual cues perpendicular to the line of progression augment the performance of persons with PD.

An order effect cannot be ruled out because the trials within each block were administered in the same order. This is especially true for the visual block where the last condition in the block had the greatest increase in pedaling rate. However, in the auditory block, we did not observe a pattern of change that could be explained by order.

Other factors that may have influenced pedaling rate include that participants may have warmed up, resulting in a faster pedaling rate over time, or, the short trial length of 1 min may not have given participants enough time to adjust to the stimulus. Future studies should include trials of longer length.

The auditory and visual blocks were not parallel comparisons. However, in designing the protocol, we were interested in the effects of optic flow without, then with, VCs in the visual condition resulting in an additional trial compared to the auditory condition. Regardless, an added trial in the auditory condition (auditory cues at baseline speed) would remedy this.

Feedback from participants as well as the investigators’ observations suggested several additions to the existing VE in order to increase engagement and promote longer-term use. These include variations in scenes and terrain, with the addition of curves and obstacles. A few participants remarked that they would have enjoyed the scene more if the road had curves in it. Obstacles embedded in the environment such as an animal crossing the road, or children playing on the side of the road would have made navigating the environment more challenging. In fact, one participant remarked that they were “…waiting for an object to pop out in front of them on the road”. For the purpose of this study however, the goal was to understand the role of visual and auditory cueing without confounding the response with other visual stimuli. The careful assessment of single features in a VE used in this study is a proposed strategy to progressively build evidence-based environments.

## Conclusion

In this study, the walking literature was adapted to cycling to determine if short-term changes in motor behavior could be achieved by embedding auditory and visual cues in a cycling VE, with the ultimate goal of promoting long-term changes to promote fitness. Our findings validate that a virtual cycling environment embedded with auditory and visual cues can modulate pedaling rate in age-matched healthy adults and persons with PD. Of clinical importance is the need to explicitly instruct persons with PD to attend to the visual cues to increase the response to the environment. This creates interaction between the clinician, patient, and VE, and indicates that VEs are not static but can be modified by the clinician by explicitly directing attention to a salient cue to modify a response.

The semi-immersive and simple environment that was created provided a strong enough stimulus to produce a response from both groups. This is important when choosing to implement this method in a clinic where space may be at a premium. In addition to the role of cueing in a cycling VE, the investigators have also assessed the role of feedback and directed attention, which complement the findings reported here.

## Abbreviations

ANOVA: Analysis of Variance
H&Y: Hoehn and Yahr
MoCA: Montreal Cognitive Assessment
PD: Parkinson’s Disease
RPM: Revolutions per Minute
UPDRS: Unified Parkinson’s Disease Rating Scale
VE: Virtual Environment
